# Targeting of the E3 ubiquitin-protein ligase HUWE1 impairs DNA repair capacity and tumor growth in preclinical multiple myeloma models

**DOI:** 10.1038/s41598-020-75499-3

**Published:** 2020-10-28

**Authors:** Viktoria Kunz, Kathryn S. Bommert, Jessica Kruk, Daniel Schwinning, Manik Chatterjee, Thorsten Stühmer, Ralf Bargou, Kurt Bommert

**Affiliations:** Comprehensive Cancer Center Mainfranken, University Hospital Würzburg, Versbacher Str. 5, 97078 Würzburg, Germany

**Keywords:** Cancer models, Cell biology, Myeloma

## Abstract

Experimental evidence suggests that ubiquitin-protein ligases regulate a number of cellular processes involved in tumorigenesis. We analysed the role of the E3 ubiquitin-protein ligase HUWE1 for pathobiology of multiple myeloma (MM), a still incurable blood cancer. mRNA expression analysis indicates an increase in *HUWE1* expression levels correlated with advanced stages of myeloma. Pharmacologic as well as RNAi-mediated HUWE1 inhibition caused anti-proliferative effects in MM cell lines in vitro and in an MM1.S xenotransplantation mouse model. Cell cycle analysis upon HUWE1 inhibition revealed decreased S phase cell fractions. Analyses of potential HUWE1-dependent molecular functions did not show involvement in MYC-dependent gene regulation. However, HUWE1 depleted MM cells displayed increased DNA tail length by comet assay, as well as changes in the levels of DNA damage response mediators such as pBRCA1, DNA-polymerase β, γH2AX and Mcl-1. Our finding that HUWE1 might thus be involved in endogenous DNA repair is further supported by strongly enhanced apoptotic effects of the DNA-damaging agent melphalan in HUWE1 depleted cells in vitro and in vivo. These data suggest that HUWE1 might contribute to tumour growth by endogenous repair of DNA, and could therefore potentially be exploitable in future treatment developments.

## Introduction

The enzyme *H*ECT, *U*BA And *W*WE Domain Containing *E*3 Ubiquitin Protein Ligase 1 (HUWE1) belongs to the *H*omologous to *E*6-AP *C*arboxyl *T*erminus (HECT) E3 ligases. HUWE1 catalyses both mono-ubiquitination and by using either K6, K11, K48 or K63 sites poly-ubiquitination of its substrates^1^. HUWE1 activity has been implicated in multiple cellular processes including, but not limited to, cell differentiation, proliferation, apoptosis, and DNA damage repair^2^. Thus, HUWE1 is critical for maintaining adult stem cell dormancy in hematopoietic stem cells^3^. In addition, B cell-specific knockout of *HUWE1* in mice demonstrated that interaction of HUWE1 with p53 is critical for both normal B cell development^4^ and the malignant growth of MYC-driven B cell cancers^5^. However, conflicting data regarding the impact of HUWE1 activity on MYC regulated genes and proliferation have been reported in colorectal cancer^6,7^.

Furthermore, HUWE1 function has also been implicated in the DNA damage response (DDR) ensuring genome integrity and comprising several different pathways specific for the respective type of DNA damage, e.g. oxidized or deaminated bases, bulky lesions such as DNA–protein adducts, or DNA double-strand breaks (DSB). Thus, HUWE1 regulates the degradation of β and λ DNA polymerases, both of which are critical for genome stability and are involved in DDR^8,9^. In addition, H2A histone family member X (H2AX), a major DDR regulator upon DSBs, is degraded upon ubiquitination by HUWE1^10^. Furthermore, HUWE1 has also been implicated in histone regulation^11^, and in the RNF8-dependent ubiquitylation response after UV- or IR-induced DSBs^12^.

Multiple myeloma (MM) remains a largely incurable B cell cancer requiring further efforts to better understand its pathobiology. Dependency on the ubiquitin–proteasome system is considered to be a hallmark of this disease affecting multiple critical cellular processes including cell cycle and apoptosis regulation, DNA damage repair or development of drug resistance^13–15^. Some of the most successful recent therapeutic approaches are targeting this system, e.g. proteasome inhibitors and cereblon-binding IMiDs. However, our current knowledge about E3 ubiquitin-protein ligases, a critical but complex component of the ubiquitin–proteasome system, and their potential role for MM pathobiology, is still limited.

Here, we ask whether HUWE1 might contribute to the malignant phenotype of MM, and investigate its role for malignant growth, MYC-dependent gene regulation and the DDR.

## Results

### *HUWE1* expression increases during MM disease progression

**Figure 1:**
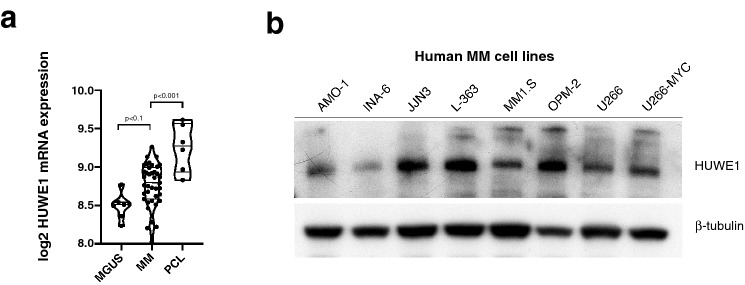
*HUWE1* mRNA and protein is expressed in primary human MM samples and HMCLs**.** **(a)** *HUWE1* mRNA expression levels increase during disease progression, MGUS (8.49 ± 0.15) intramedullary (8.77 ± 0.27) and extramedullary MM cells (9.25 ± 0.28 log2 expression value). Expression analysis based on a publicly available database (GSE2113; n = 52 samples). The significance level was calculated according to the two-tailed Student's t-test between MGUS/MM cells and MM/PCL cells. **(b)** Western blot analysis of HUWE1 expression in HMCLs, β-tubulin served as loading control.

First, we analysed *HUWE1* expression in pre-malignant and malignant plasma cells (PCs) exploiting the publicly available GSE2113 mRNA data set^16^. Compared to pre-malignant PCs from patients with monoclonal gammopathy of undetermined significance (MGUS), *HUWE1* mRNA expression levels were significantly increased in bone marrow-derived MM cells, and still higher in leukemic MM cells (Fig. 1a). Additionally, a similar analysis using the GSE6477 dataset, which includes bone marrow-derived normal PCs and PCs from MGUS, Smoldering MM and newly diagnosed MM, also showed the same tendency (Supplementary Fig. 1a). These data indicate that *HUWE1* expression is higher in advanced stages of MM. Western blot analysis confirmed HUWE1 expression in all human MM cell lines (HMCLs) tested (AMO-1, INA-6, JJN3, L-363, MM1.S, OPM-2, U266 and U266-MYC (Fig. 1b).

### Antitumor effects of the pharmacologic HUWE1 inhibitor BI8622

**Figure 2:**
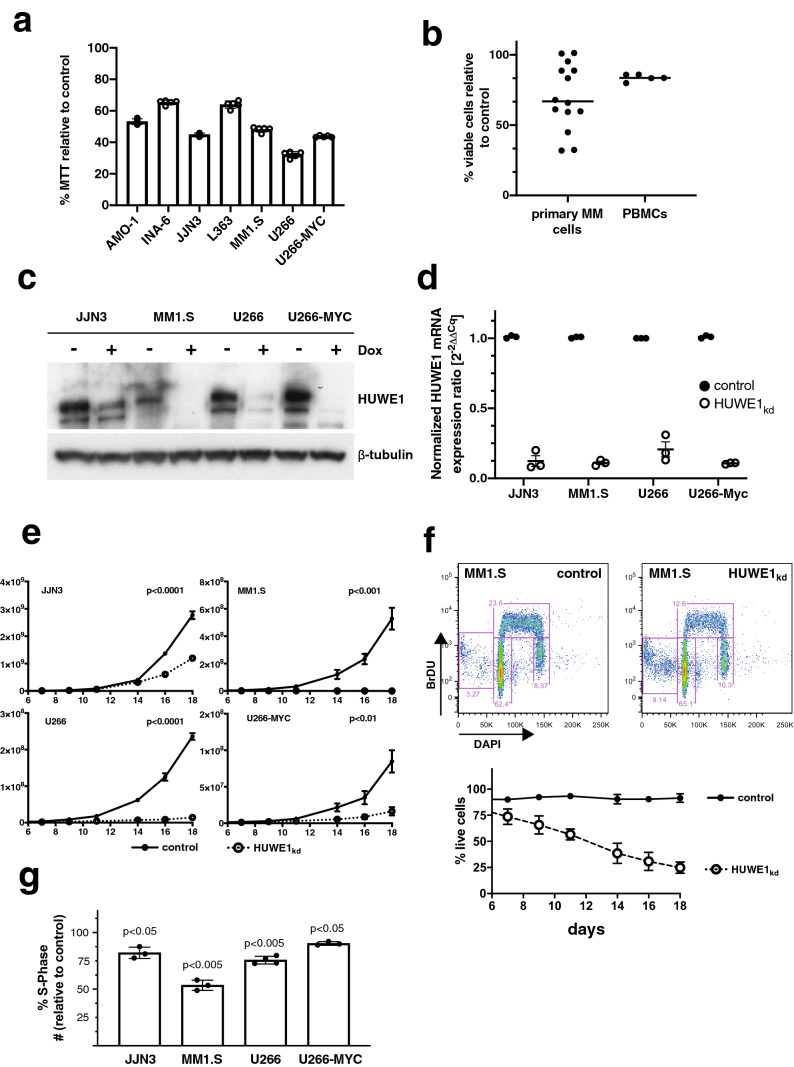
Inhibition of HUWE1 activity by the pharmacologic inhibitor BI8622 or doxycycline-dependent shRNA-mediated *HUWE1* knockdown.** (a)** Inhibition of proliferation in HMCLs with small molecule HUWE1 inhibitor BI8622. Cells were incubated for 48 h with 10 µM BI8622 and the reduction of MTT relative to the solvent-treated control was used as proliferation marker. The results (mean with standard deviation) were as follows: AMO-1 (53.15 ± 1.78), INA-6 (65.34 ± 1.39), JJN3 (44.86 ± 0.1), L363 (63.95 ± 2.32), MM1.S (48.20 ± 1.66), U266 (32.20 ± 1.97), and U266-MYC (43.71 ± 0.55). **(b)** Effect of BI8622 on primary human MM samples and PBMCs measured after 48 h incubation with 10 µM BI8622. Percentage of viable cells using annexin V-FITC and PI staining was calculated relative to respective solvent-treated controls. **(c)** Western blot analysis of doxycycline-induced (+ Dox, 1 µg/ml) downregulation of HUWE1 protein at day 5 post-induction. β-tubulin served as loading control. **(d)** Quantification of *HUWE1* mRNA expression after HUWE1 knockdown (black circle: control, white circle: doxycycline induced *HUWE1* knockdown), after 5 days the normalized expression relative to control was 0.123 ± 0.038 for JJN3, 0.11 ± 0.012 for MM1.S, 0.207 ± 0.054 for U266, and 0.107 ± 0.003 for U266-MYC. **(e)** Decreased proliferation upon *HUWE1* knockdown in HMCLs. Cells were counted every 3 days and cultivated at the same density. Solid line: control, dotted line: *HUWE1* knockdown. **(f)** FACS-based cell cycle analysis by BrdU/DAPI staining upon HUWE1 knockdown in MM1.S cells. Shown is a representative experiment with histogram and gating of the cell cycle fractions (sub G1, G1, S, G2). Viable (trypan blue negative) MM1.S cells were counted over the course of 18 days in the *HUWE1* knockdown and control cultures. **(g)** Quantification of the S phase between control and *HUWE1* knockdown cells. Shown are the mean and standard deviation of 3 independent experiments in JJN3, MM1.S, U266 and U266-MYC cells.

BI8622 has previously been described as an inhibitor of HUWE1 activity^7^. In order to investigate whether malignant growth of MM cells might be affected by pharmacologic HUWE1 inhibition, we treated HMCLs (Fig. 2a), primary MM cells and normal mononuclear cells from peripheral blood (PBMCs; Fig. 2b) with 10 µM BI8622 for 48 h prior to viability analysis by MTT assay (HMCLs) or annexin V/PI staining and FACS measurement (primary MM cells and PBMCs). BI8622 treatment led to significantly lower viability in all HMCLs tested (Fig. 2a), a dose response curve for MM1.S and BI8622 is shown in supplementary Fig. 2b. Primary MM cells obtained from 14 different patients showed heterogeneous sensitivity towards BI8622 treatment, with a more sensitive subgroup (n = 8) distinguishable from a group of rather insensitive samples (n = 6) (Fig. 2b). There was no correlation between the response towards BI8622 and the clinical parameters from the patients (Supplementary Table 1). In contrast, treatment of PBMCs with BI8622 did not lead to substantial loss of viability (n = 5) (Fig. 2b). These results indicate that HUWE1 might support MM cell growth/survival in vitro. Unfortunately, however, the short serum half-life of BI8622 precludes its use in in vivo models.

### Sustained inducible knockdown of *HUWE1* strongly reduces proliferation of HCMLs in longer-term culture

We therefore decided to also analyse the role of HUWE1 in MM by molecular genetic approaches and generated sublines of JJN3, MM1.S, U266 and U266-MYC stably transfected with a doxycycline-inducible shRNA expression cassette targeting *HUWE1*. Western blot analysis revealed effective depletion of HUWE1 protein in all HMCL sublines 5 days after addition of doxycycline to the cell culture (Fig. 2c). Accordingly, real time qPCR analysis demonstrated about 80–90% decreases of *HUWE1*-mRNA levels (Fig. 2d). Over the 18-day study period HUWE1 knockdown strongly affected the growth curves of all MM cell lines tested, although to different degrees (Fig. 2e). Whereas HUWE1 knockdown in JJN3 cells resulted in a slight increase of their doubling time from 1.51/1.82 days (95% CI) to 1.80/2.13 days, the effect in MM1.S cells was a shift from 1.41/2.23 days to 13.06 days/infinity. Analysis of the live cell fraction in the treated cultures showed only in MM1.S a decrease of viable cells, whereas in JJN3, U266 and U266-MYC no decrease of viability could be detected (Fig. 2f and supplementary Fig. 4a). Doxycycline treatment of parental cells was without discernible effect over the same time frame (shown for MM1.S and U266 cells in Supplementary Fig. 1c). U266 cells, which lack endogenous MYC expression, were more sensitive to HUWE1 knockdown (1.90/2.20 days vs 3.38/4.17 days) than MYC overexpressing U266-MYC cells (1.37/2.37 vs 1.69/4.22 days). This relative resilience of the U266-MYC growth curve was intriguing as HUWE1 has previously been reported to activate MYC target gene expression via MIZ1 inhibition in colon cancer cell lines^7^. However, of our four inducible *HUWE1* knockdown models only the MYC negative U266 cell line displayed slight HUWE1 dependence of mRNA levels of MYC-dependent genes (*RPL29*, *RPL37*; Supplementary Fig. 1b), and altered MIZ1 expression levels upon HUWE1 knockdown were not observed at all (supplementary Fig. 3a). We therefore conclude that the effects of HUWE1 depletion on MM cell growth are not primarily mediated via MYC and MIZ1. To further characterize the observed anti-proliferative effect upon *HUWE1* knockdown, we used bromodeoxyuridine (BrdU) and 4′,6-diamidino-2-phenylindole (DAPI) staining for FACS-based cell cycle analysis. *HUWE1* knockdown entailed a significant reduction of the S phase in all HMCLs tested (Fig. 2f,g; supplementary Fig. 4a). In MM1.S we detected a decrease of viable cells over the analysed period of 18 days and an increase of the sub-G1 fraction in the cell cycle analysis.

**Figure 3:**
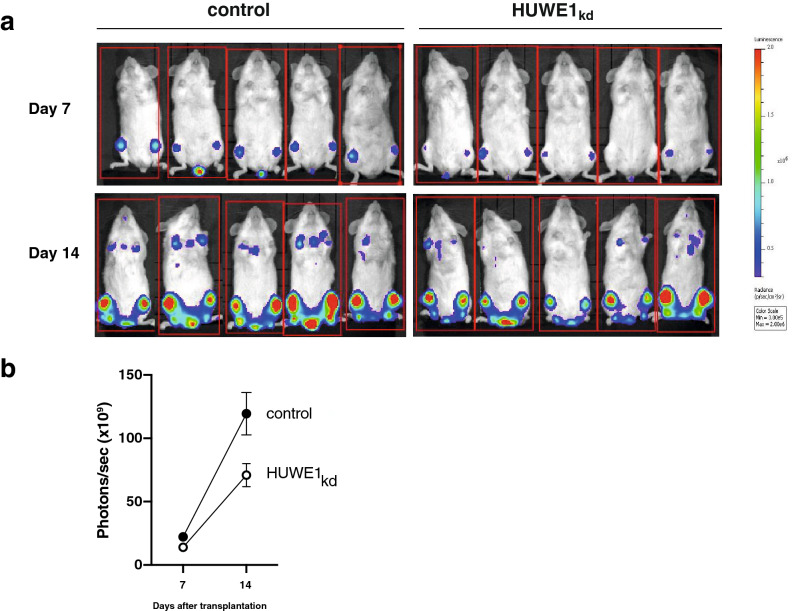
*HUWE1* knockdown reduces tumour growth of xenotransplanted MM1.S-shHUWE1 cells in NSG mice in vivo. **(a)** In vivo imaging of tumour load 7 and 14 days after MM1.S cell transplantation. The cells were injected into the tail vein of the animals, and they were separated into control (n = 5) and *HUWE1*_kd_ groups (n = 5). *HUWE1* knockdown was induced with doxycycline (200 mg/kg) containing diet. (**b)** Tumor growth was measured by counting emitted photons/sec in the animals without (*HUWE1*_kd_, white symbol) and with HUWE1 expression (control, black symbol) in the luciferase expressing MM1.S cells after i.p. luciferin injection. Images were recorded using Living Image software, version 4.4 (Perkin Elmer, Waltham, USA).

**Figure 4:**
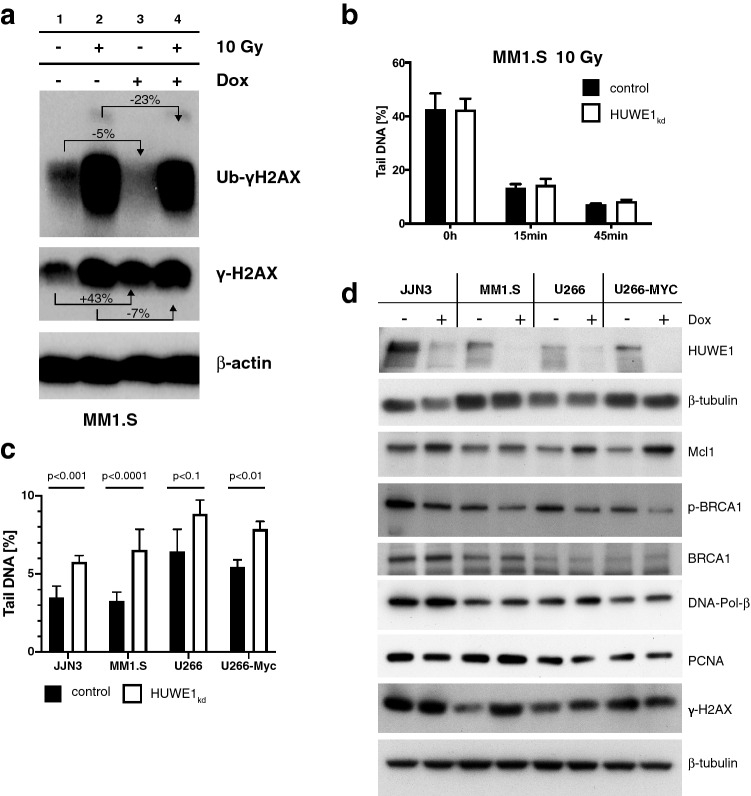
Role of HUWE1 in DNA repair.** (a)** HUWE1 knockdown (+ Dox) reduces ubiquitinated γH2AX (Ub-γH2AX) steady state level (lane 1 vs lane 3). Radiation (10 Gy) induced strong expression of γH2AX and Ub-γH2AX, less pronounced in the absence of HUWE1. (**b)** *HUWE1* knockdown does not significantly alter the repair of DNA double strand breaks after irradiation of MM1.S cells with 10 Gy, as measured by the Comet assay over 45 min. (**c)** *HUWE1* knockdown significantly increased endogenous DNA damage as measured by DNA tail length in a Comet assay (black bars: control, white bars: HUWE1_kd_. Means and standard deviations: 3.51 ± 0.41% to 5.79 ± 0.23% for JJN3, 3.28 ± 0.23% to 6.54 ± 0.54% for MM1.S, 6.45 ± 0.70% to 8.84 ± 0.44% for U266, and 5.46 ± 0.26% to 7.88 ± 0.28% for U266-MYC cells. Cells were harvested either 5 days (MM1.S) or 7 days post-induction. (**d)** Western blot analysis of DDR components in four HCMLs without (− Dox) and with (+ Dox) doxycycline-induced *HUWE1* knockdown (+ Dox). All HMCLs studied showed an increase in Mcl1 and decreased phosphorylated BRCA1 (pBRCA1) levels after HUWE1 knockdown. No changes could be detected for BRCA1, PCNA, DNA-Pol-β. A change in γH2AX could only be detected in MM1.S. β-tubulin served as loading control.

### *HUWE1* knockdown inhibits malignant MM growth in the MM1.S/NOD scid mouse model in vivo

Next, we asked whether HUWE1 might also support malignant growth of MM cells in vivo. For these experiments, MM1.S cells, stably transfected to express luciferase for bioimaging, and harbouring a doxycycline-inducible shRNA expression cassette targeting HUWE1, were injected into the tail veins of NOD scid gamma (NSG) mice (Fig. 3a). Prior to MM cell inoculation, the mice were divided into a control group which was fed a normal grain-based diet, and a test group which received a diet containing doxycycline (200 mg/kg). Notably, in the immediate time period following injection, when MM1.S cells migrate into the bone marrow, they still express HUWE1 because it takes about 3 to 4 days of exposure to doxycycline to implement full-scale HUWE1 knockdown (Fig. 2 and supplementary Fig. 2c). At day 7 post-inoculation the control group displayed significantly larger tumour mass than the HUWE1 knockdown group (22.14 × 10^9^ ± 4.70 × 10^9^ vs. 13.98 × 10^9^ ± 5.61 × 10^9^ photons/sec). This difference was further magnified at 14 days post injection, when the control group was at 119.38 × 10^9^ ± 37.69 × 10^9^ photons/sec and HUWE1 knockdown group at 70.92 × 10^9^ ± 20.35 × 10^9^ photons/sec (Fig. 3b). These data demonstrate that HUWE1 loss reduces tumour growth in vivo.

### Loss of HUWE1 expression impairs DNA repair capacity

Based on its reported involvement in DDR regulation, we hypothesized that the observed effects of *HUWE1* knockdown on cell cycle regulation and proliferation are functionally linked to its role in DDR^2,17^. First, we assessed the effects of HUWE1 depletion on ubiquitination of γH2AX which has been shown to be important for both DSB signalling and restarting of stalled replication forks^10,17^. After irradiation of MM1.S cells (10 Gy) both γH2AX and ubiquitinated γH2AX increased to slightly lesser extents in HUWE1 knockdown cells (Fig. 4a), but this still constituted functional DSB repair signalling. Similarly, HUWE1 knockdown did not affect the capacity for DNA repair after acute irradiation damage, as measured by the % DNA tail-length in a Comet assay over a 45-min time-window, in any HMCL tested (exemplarily shown for MM1.S cells; Fig. 4b). However, we also observed that the steady state level of ubiquitinated γH2AX was decreased in MM1.S cells in the absence of HUWE1 expression, whereas γH2AX was increased (Fig. 4a, lanes 1 and 3). Next, we therefore analysed the steady state levels of DNA tail-length in different HMCLs with or without prolonged HUWE1 knockdown and found significant increases in all HUWE1-depleted conditions, indicative for increased endogenous DNA damage (Fig. 4c). Finally, we analysed a number of regulatory factors known to interact with HUWE1 and to be associated with DDR (e.g. γH2AX, BRCA1, Mcl1, p53, DNA-Polymerase β^8–10,17^) by Western blotting of MM cells with and without HUWE1 depletion (Fig. 4d). Whereas a strong increase of γH2AX was found in MM1.S cells (the HMCL most sensitive for growth inhibition by HUWE1 knockdown), this effect was less pronounced in U266 and absent in JJN3 or U266-MYC cells. In contrast, phospho-BRCA1 levels decreased and Mcl-1 expression increased upon HUWE1 knockdown in all HMCLs analysed (Fig. 4d). In contrast to other reports, we found no consistent evidence that HUWE1 is involved in the degradation of H2AX, HDM2, p53 or p21 (supplementary Fig. 2c), or of PCNA, BRCA1 or DNA-Pol β (Fig. 4d). Indeed, in the HMCLs tested the levels of DNA-Pol β were slightly increased upon HUWE1 knockdown (Fig. 4d). Taken together, these data indicate that the endogenous DNA damage repair is—at least in functionally distinguishable parts—negatively affected by the absence of HUWE1.

### HUWE1 knockdown strongly enhances growth-inhibitory effects of the DNA-damaging anti-MM agent melphalan in the MM1.S/NSG mouse model

**Figure 5:**
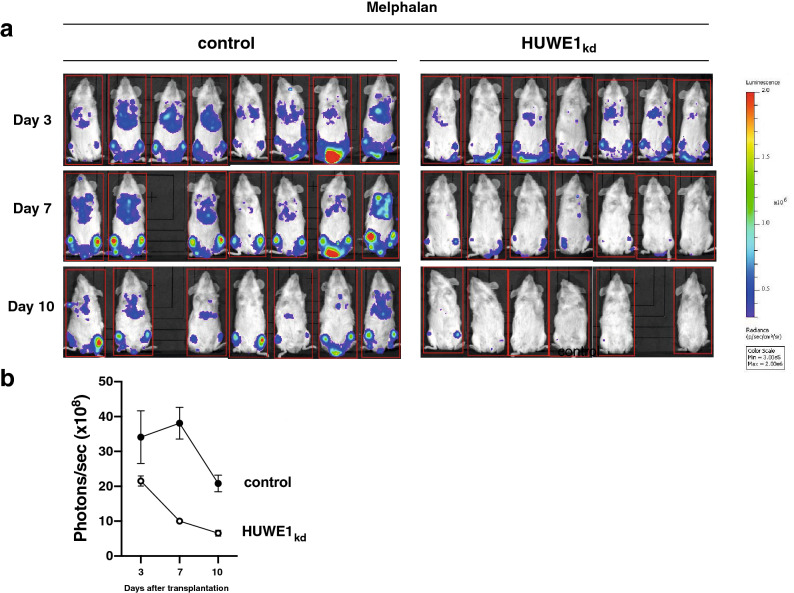
*HUWE1* knockdown enhances the anti-tumour effect of melphalan in the MM1.S/NOD scid mouse model in vivo. **(a)** In vivo imaging of tumour load 3, 7 and 10 days after MM1.S cell transplantation. *HUWE1* knockdown was induced with a doxycycline (200 mg/kg) containing diet. Both *HUWE1*_kd_ (n = 7) and control animals (n = 8) received melphalan (5 mg/kg) every 3 days by i.p. injection. (**b)** Tumor growth was measured by counting emitted photons/sec in the animals without (*HUWE1*_kd_, white symbols) and with *HUWE1* expression (control, black symbols) in the luciferase expressing MM1.S cells after i.p. luciferin injection. Images were recorded using Living Image software, version 4.4 (Perkin Elmer, Waltham, USA).

Melphalan, an established chemotherapeutic agent in the treatment of MM patients, damages DNA which, if not repaired, leads to induction of apoptosis in HMCLs (for review^18^). Therefore, we investigated if HUWE1 depletion may potentiate the anti-myeloma activity of melphalan. In the presence of 2.5 µm melphalan, knockdown of HUWE1 decreased proliferation of MM1.S cells in vitro from 69.41% ± 5.67 to 35.04% ± 1.24 compared to control as measured by MTT (Supplementary Fig. 4b). We then asked, if the increased melphalan sensitivity in vitro would also translate into an enhanced anti-tumour effect in vivo. Two groups of NSG mice (either with or without doxycycline-containing diet) were injected i.v. with luciferase expressing MM1.S cells endowed with an inducible expression cassette for shRNA targeting HUWE1 (Day 0), and the cells were allowed to migrate to the bone. Thereafter, starting at day 3 all mice were injected i.p. with 5 mg/kg melphalan (at days 3, 6 and 9), and bioluminescencent imaging was performed at days 3, 7 and 10 (Fig. 5a). Concomitant melphalan application and HUWE1 knockdown resulted in robust suppression of tumour growth (at day 7 post knockdown induction: 10.00 × 10^8^ ± 1.12 × 10^8^ photons/sec vs 38.09 × 10^8^ ± 12.07 × 10^8^ photons/sec in the melphalan-only control group; Fig. 5a,b). Moreover, this inhibition continued over 10 days (6.57 × 10^8^ ± 2.02 × 10^8^ photons/sec without HUWE1 and 20.81 × 10^8^ ± 6.30 × 10^8^ photons/sec with HUWE1) until the termination of the experiment due to animal health. These data support our finding that HUWE1 is involved in endogenous DNA damage repair which represents a salvage mechanism for DNA-damaged cells after treatment with alkylating agents like melphalan.

## Discussion

Therapeutic intervention targeting molecular machineries that involve the ubiquitin proteasome system has been central to recent clinical advances in MM treatment and now forms the bedrock of current MM therapies^15^. However, many functional aspects of ubiquitination, in particular its role in tumorigenesis and as a potential therapeutic target remain to be better explored. Here we have investigated the HECT-E3 ligase HUWE1, which has been implicated in a number of oncogenic processes such as MYC regulation^6,7^ and DNA damage response (DDR)^6,12,17^, for its role in multiple myeloma. *HUWE1* expression at either RNA- or protein level was detected in every MM cell line tested, which is in accordance with its pattern of ubiquitous expression in either normal or cancer tissue (www.protein-atlas.org/ENSG00000086758-HUWE1/pathology). Analysis of *HUWE1* mRNA expression data from plasma cells also showed a moderate but still marked tendency for increase from the pre-malignant stages (normal PCs, MGUS) through intramedullary MM to plasma cell leukaemia suggesting a pathogenetic involvement of HUWE1 during MM progression to more aggressive forms. Inhibition of HUWE1 activity either by the pharmacologic compound BI8622 or by shRNA-mediated knockdown led to decreased growth and/or viability in MM cell lines as well as in a substantial fraction of primary MM samples, supporting a potential role of HUWE1 for malignant expansion. Both approaches varied somewhat in the extent of their effects and the pronounced decrease in viability across the board in MM cell lines after incubation with 10 µM BI8622 may in parts be due to different on-target effects to paralogs of HUWE1, e. g. HECTD4, or to off-target effects. However, the knockdown approach also demonstrated that HUWE1 depletion entails reduced proliferation rates, as shown by reduced S-phase components in cell cycle analyses. Accordingly, application of inducible *HUWE1* knockdown in an MM1.S xenotransplantation model showed for the first time that blockade of HUWE1 expression is also effective at slowing tumour growth in vivo. Our data thus support findings in other cancer entities showing a role for HUWE1 for malignant growth^5–7^.

It was therefore surprising that neither in MM cell lines engineered for doxycycline-inducible HUWE1 knockdown nor in the MYC-overexpressing subline U266-MYC, moderate to strong HUWE1 depletion had any discernible effects on the expression levels of MYC target genes, nor on MIZ protein, an inhibitor of MYC activity. Unlike its role in colon cancer, our data thus do not imply HUWE1 as important regulator of MYC activity in MM. However, our results are compatible with recent in-depth analyses of the genetic mechanisms of MYC dysregulation in MM, which suggest that—unlike in other cancer entities—*MYC* expression is not correlated with proliferation but may rather support other physiological needs of plasma cell tumors ^19^.

Different authors have implicated HUWE1 function in the DNA damage response. Interestingly, although we found no impairment of the immediate response to irradiation-induced acute DNA damage in HUWE1 depleted MM cells, we show for the first time that prolonged *HUWE1* knockdown always led to significant accumulation of damaged DNA as witnessed by Comet-assay, clearly indicating a role for HUWE1 in the response to endogenous DNA damage. Yet, only in MM1.S cells, this effect was translated into a notable increase in γH2AX levels. At this point, it remains unclear if absence of increased levels of γH2AX in HUWE1-depleted cells might be a result of general impairment of the detection of damaged DNA, or if only in MM1.S cells single strand breaks are converted to the double strand breaks that induce formation of γH2AX.

Amongst a host of potential DDR mediators analysed, increased levels of Mcl1 and DNA polymerase β and decreased levels of phospho-BRCA1 were the only changes consistently observed in HUWE1-depleted MM cells. Mcl1 has indeed recently been shown to be targeted for degradation by HUWE1^20^, but a connection to impaired DDR has so far only been shown in the context of Mcl1 depletion^21^. In addition it has been reported that Mcl1 acts as a functional switch between NHEJ and HR DNA repair pathways^22^. Likewise, the functional relationship between HUWE1 and BRCA1 remains puzzling, because in contrast to breast cancer cells^23^, HUWE1 depletion did not affect levels of BRCA1 itself. Similar to the absence of increased γH2AX, though, the strong decrease of phosphorylated BRCA1 again implies defective detection of DNA damage or impaired initiation of DNA damage responses in HUWE1-depleted cells. Our suggestion is that the BRCA1 phosphorylation may be facilitated by a ubiquitin tag from HUWE1, but that this modification is not essential for DDR after IR exposure.

Recently, it was demonstrated that inhibition of nucleotide excision repair increases sensitivity of HMCLs to the DNA-alkylating anti-MM agent melphalan^24^. Because the observed antiproliferative effects of HUWE1 depletion in MM cells also appear to be mediated by impaired repair of endogenous DNA damage, it was rational to test melphalan effectivity within the context of HUWE1 knockdown. While this combination only produced a modest effect on viability in vitro, MM tumour growth in vivo continually decreased over the length of the study indicating that knockdown of HUWE1 sensitized the cells to melphalan-induced cell death. Genome-wide mutation analysis in newly diagnosed MM patients identified driver mutations in *HUWE1*. The majority of the reported mutations affect splice sites in the *HUWE1* mRNA with the consequence of HUWE1 inactivation^25^. In light of our data, these would suggest, that MM patients with HUWE1 inactivation display a reduced DNA repair capacity. Therefore HUWE1 would act as a tumor suppressor assisting in DNA repair in MM cells which due to the high replicative stress display increased DNA damage^26,27^.

In summary, our data support a role for HUWE1 in the regulation or execution of endogenous and melphalan-induced DDR, and in this capacity may contribute to the malignant phenotype of MM. Pharmacological targeting of HUWE1 could be an attractive option to increase effectivity of MM therapies mechanistically related to induction of DNA damage.

## Methods

### Cell culture

The human MM cell lines (HMCLs) U266 and JJN3 were purchased from the German Collection of Microorganisms and Cell Cultures (DSMZ, Braunschweig, Germany), MM1.S was bought from LGC Biolabs (Wesel, Germany), and the stably c-MYC-expressing U266-MYC subline was established in our laboratory as previously described^28^. All HCMLs were cultivated in RPMI-1640 medium supplemented with 10% fetal bovine serum (FBS), GlutaMAX, 1 mM sodium pyruvate, and 100 µg/ml gentamicin (AppliChem, Darmstadt, Germany).

### Primary MM cells

Primary MM cells were obtained from routine diagnostic bone marrow aspirates of patients after informed consent with permission of the Ethics Committee of the University of Würzburg (76/13). The primary MM cells were purified from the bone marrow aspirate by CD138 MicroBeads (Miltenyi) as previously described^29^. Primary MM cells were kept in RPMI-1640 supplemented with 10% FBS, 10 mM HEPES, 1 mM sodium pyruvate, 2 mM L-glutamine, 2 mg/l glucose, 100 µg/ml gentamicin and with 2 ng/ml interleukin 6^30^.

### Cloning of inducible HUWE-1 shRNA and of firefly luciferase lentiviral vectors

pHAGE-dsRed-firefly luciferase was cloned by digesting pHAGE-CMV-DsRed-UBC-eGFP (addgene #24526) and replacing eGFP with PCR amplified firefly luciferase. Linearized pHAGE and fragments were ligated using Gibson Assembly Master Mix (NEB). pINDUCER11-shHUWE was cloned by inserting a published shRNA encoding sequence for HUWE1 into pINDUCER-11^7,31^ using Gibson Assembly (NEB).

### Lentivirus production and HMCL transduction

Lentivirus production and transduction of MM cell lines was performed as previously described^28^.

### Western blot

Protein expression analysis was performed as previously described^32^. Cells were lysed in RIPA-buffer (Sigma-Aldrich, R0278) supplemented with proteinase inhibitor cocktail (AppliChem, A7779). Samples with 15 µg (or 30 µg for HUWE detection) were separated by SDS-PAGE and transferred onto Immobilon-P PVDF membranes (Millipore). Membranes were incubated for 1 h at room temperature or overnight at 4 °C in antibody solution with 1–5% BSA or 5% non-fat dry milk/TBS-T (20 mM Tris (pH 7.4), 0.5 M NaCl, 0.1% Tween-20). Antibodies used in this study are listed in supplementary information.

### Growth study

2 × 10^5^ cells/ml were plated in 25 cm^2^ flasks containing 10 ml RPMI-1640 GlutaMAX with 10% FBS, 1 mM sodium pyruvate, 100 µg/ml gentamicin and 1 µg/ml doxycycline. Every 2–3 days cells were washed in PBS and cell numbers were determined by a trypan blue (Sigma-Aldrich, T8154) exclusion method using a haemocytometer and by FACS (CyFlow SL, Partec).

### BrdU cell cycle analysis

Cells were treated with 10 µM BrdU (BioLegend) for 2 h. After washing twice with PBS, the cells were fixed in ice-cold 70% ethanol for 30 min at 4 °C. The cells were resuspended in 1 ml freshly prepared 2 M HCl/0.5% Triton X-100/PBS and incubated for 20 min at room temperature. After washing twice with PBS, the cells were treated with 1 ml 0.1 M sodium tetraborate/0.5% Tween/PBS for 10 min at room temperature. The cells were washed with PBS and incubated in 100 µl antibody solution (0.4 µl APC-conjugated anti-BrdU antibody, BioLegend in PBS with 5% FBS) for 30 min at room temperature. After washing, the cells were treated with 50 µl RNase A (10 µg/ml) for 30–60 min. Finally, cells were stained with 3.3 µg/ml DAPI for 10 min. Flow cytometry was performed with a FACS Canto II (BD BioSciences) and data were analyzed by FlowJo V9.

### MTT assay

Cell sensitivity to melphalan with and without HUWE1 depletion was measured using an MTT-based assay. Metabolic activity was quantified by adding 10 μl Thiazolyl Blue Tetrazolium Bromide (final concentration 0.5 mg/ml) (Sigma-Aldrich). After 4 h 100 μl solubilization solution (10% SDS in 0.01 M HCl) was added and after overnight incubation at 37 °C absorbance of the solubilized reduced formazan was measured at 570 nm with a microtiter plate reader (Sunrise, Tecan).

### Alkaline single-cell electrophoresis “Comet Assay”

Detection and quantification of DNA damage was assayed using the Comet Assay essentially as described^33^.

### qPCR, real time PCR

RNA extraction was performed using the NucleoSpin RNA isolation kit (Macherey–Nagel). cDNA was synthesized from 1 μg total RNA using the High-Capacity cDNA Reverse Transcription Kit (Thermo Fisher Scientific). Each cDNA sample was pre-diluted 1:20 and 4 μl were used with 4 × Luminaris Color HiGreen qPCR Master Mix (Thermo Fisher Scientific) and 250 nM primer for the qPCR reaction. A two-step cycling protocol was performed as follows: UDG pre-treatment for 2 min (50 °C), initial denaturation for 10 min (95 °C), 40 cycles of 15 s at 95 °C, 40 s at 60 °C (CFX Connect Thermal Cycler, Bio-Rad). The relative quantity of the target mRNA was normalized to beta-2-microglobulin). The fold changes in RNA expression were calculated using the 2^−ΔΔCt^ method^28^. Primer sequences are listed in supplementary information.

### Animal work

All experiments were performed with female NOD.Cg-Prkdc^scid^ Il2rg^tm1Wj^I/SzJ (NSG)-mice (Charles River, Sulzfeld) according to the German regulation for animal experimentation. 1 × 10^6^ MM1.S-shHUWE1-firefly luciferase cells were washed, suspended in RPMI-1640 GlutaMAX medium and injected intravenously into NSG mice. Animals in the HUWE_kd_ group received a doxycycline-supplemented diet (A112-D00203, ssniff, Soest). For the melphalan treatment animals were injected with 5 mg/kg melphalan (Sigma-Aldrich, M2011) on days 3, 7 and 10 after MM1.S cell transplantation. The growth of MM1.S cells was documented by bioluminescence imaging, IVIS Lumina system (PerkinElmer, Waltham, MA). Mice were anesthetized i.p. with a mixture of ketamine (100 mg/kg) and xylazine 2% (10 mg/ml) in 0.9% NaCl. 0.3 g/kg D-luciferin (BioSynth, L-8220) was coinjected. 10 min after injection bioluminescence was recorded. Data were analysed using Living Image software 4.4, Perkin Elmer.

### Statistical analysis

A two-tailed Student’s t-test was applied to perform statistical analysis. Results were considered significant at p < 0.05. All experiments were performed as independent biological replicates at least three times. Calculations were performed with Prism GraphPad 8.0. All data are presented as mean ± SD.

### Ethics declaration

All animal experiments were carried out in accordance with relevant guidelines and regulations and approved by the Regierung von Unterfranken AZ 55.2 2532-2-335.

Primary human MM cells were obtained from routine diagnostic bone marrow aspirates of patients after informed consent. All experimental protocols were approved by the Ethics Committee of the University of Würzburg (AZ 76/13) and carried out in accordance with relevant guidelines and regulations.

## Supplementary information

Supplementary Information.

## Data Availability

No datasets were generated or analysed during the current study.
