# Supplementary material for: Targeting of the E3 ubiquitin-protein ligase HUWE1 impairs DNA repair capacity and tumor growth in preclinical multiple myeloma models
Source: Sci Rep. 2020 Oct 28;10:18419. doi: 10.1038/s41598-020-75499-3 (PMC7595222; doi:10.1038/s41598-020-75499-3)

Supplementary data for

Targeting of the E3 ubiquitin-protein ligase HUWE1 impairs DNA repair capacity and tumor growth in preclinical multiple myeloma models

Viktoria Kunz*, Kathryn S. Bommert*, Jessica Kruk, Daniel Schwinning, Manik Chatterjee, Thorsten Stühmer, Ralf Bargou[#] and Kurt Bommert[#]

Primers (5´-3´)

Gene	Forward Primer	Reverse Primer
RPL29:	ACACACAACCAGTCCCGAAA	TCTTGGCAAAGCGCATGTTG
RPL37:	AGCGAGATGACGAAGGGAAC	TCATTCGACCAGTTCCGGTG
B2M:	TGACTTTGTCACAGCCCAAGA	CGGCATCTTCAAACCTCCAT
MYC:	TGAGGAGACACCGCCCAC	CAACACGATTTCTTCCTCATCTTC
HUWE1:	CCAGAAGTTCTTCTTGAGGGTAC	GCCTAAACCGGAGGAACC

Firefly luciferase:

Forward: CTTGGCCGCCCATAGCGGCCGCACCATGGAAGATGCCAGAACATC

Reverse: GTAATCCAGAGGTTGATTAGGATCTATCGGCGGCCGCTTTACTTGCCGCCTTTCTTG

Antibodies used in this study (name, clone, supplier)

mouse anti- β-tubulin, BT7R, Thermo Fisher Scientific

rabbit anti Lasu1/Ureb1 (HUWE1), polyclonal, Bethyl Laboratories

rabbit anti c-Myc, Y69, abcam

mouse anti l-Myc, MycL 1-1A3, Developmental Studies Hybridoma Bank University of Iowa

mouse anti BRCA, I-20, Santa Cruz Biotechnology

rabbit anti phospho-BRCA1, polyclonal, Cell Signaling

mouse anti Chk-1, monoclonal, Santa Cruz Biotechnology

mouse anti PCNA, PC10, Cell Signaling

rabbit anti Ubiquityl-Histone H2A, D27C4, Cell Signaling Technology

rabbit anti phospho-Histone H2A.X, 20E3, Cell Signaling Technology

rabbit anti Mcl-1, polyclonal, Cell Signaling Technology
rabbit anti Miz-1, polyclonal, abcam
mouse anti p53, 1C12, Cell Signaling Technology
β-Actin HRP, 13E5, Cell Signaling Technology
rabbit anti DNA Polymerase beta, polyclonal, abcam
anti-mouse IgG-HRP, Cell Signaling Technology
anti-rabbit IgG-HRP, Cell Signaling Technology

Supplementary figure 1. **a)** *HUWE1* mRNA expression levels in normal plasma cells, and plasma cells from MGUS, Smoldering MM and newly diagnosed MM patients. Expression analysis based on a publicly available database (GSE6477; n=129 samples). **b)** qPCR analysis of MYC target genes RPL29 and RPL37 indicate that *HUWE1* knockdown (control: black symbols, $HUWE1_{kd}$: white symbols) does not alter MYC-mediated gene expression. **c)** Proliferation of MM1.S or U266 cells in the absence or presence of 1 *μ*M doxycycline for 18 days.

Supplementary figure 2

a) HMCLs were incubated with control treatment, 2.5 *μ*M melphalan, 10 *μ*M BI8622, or with both drugs for 72 hours. The cell viability was detected by MTT assay. The significance was calculated according to two-tailed Student's test (*p<0.05, **p<0.005, ***p<0.0005), indicating a stronger effect for combination treatment with melphalan and BI8622 in all cell lines tested. **b)** BI8622 dose-response in MM1.S cells. Cells were treated with 0 - 10 μM BI8622 for 72 hours. The MTT percentage indicated cell viability. Each data point represents four independent experiments (+/-SD).

c) p53, MDM2 and p21 protein levels after *HUWE1* knockdown in MM1.S cells, 10 *μ*M Nutlin incubation for 24 hours served as control for MDM2 inhibition and p21 and p53

upregulation. Extracts were prepared after 3, 5, and 7 days of doxycycline application (1 μg/ml). HUWE1 Western blot shows down regulation of HUWE1 protein after 3, 5, and 7 days of doxycycline application.

Supplementary figure 3

a) Western blot analysis of HUWE1 and CHK-1 protein expression in HMCLs before and after doxycycline-inducible *HUWE1* knockdown. MM1.S, U266, and U266-MYC showed a sharp decrease of HUWE1 protein level after DOX treatment. The *HUWE1* knockdown in JJN3 cells was less efficient. The CHK-1 protein expression is increased in MM1.S and U266 cell line but stayed unchanged in U266-MYC and decreased in JJN3 cells. β-actin served as a loading control. **b)** Western blot analysis of Mcl1, p-BRCA-1, Miz-1, and γ-H2AX protein expression in HMCLs after +/- 10 μM BI8622 treatment for 24 hours. All HMCLs studied showed an unchanged Mcl1 protein expression level. Comparable to the HMCLs with inducible shRNA expression against *HUWE1*, decreased phosphorylated BRCA1 (pBRCA1) and γH2AX levels were detected after BI8622 treatment. Miz-1 expression was only increased in MM1.S after HUWE1 inhibition while in JJN3, U266, and U266-MYC the expression stayed unchanged. β-actin served as a loading control.

Supplementary figure 4.

a) Shown are FACS-based cell cycle analyses upon BrdU/DAPI staining of control and HUWE1-depleted JJN3, U266 and U266-MYC cells and the percentage of living cells over the experimental time period, with *HUWE1*$_{kd}$ (open circle and control closed circle) **b)** MM1.S cells were incubated with or without 2.5 μM melphalan after HUWE1 knockdown or control treatment. and differences in viability determined by MTT assay. Melphalan-induced antiproliferative effects are stronger in the absence of HUWE1.

Supplementary table 1.

Patient characteristics/treatment history of the used primary patient samples.

Supplementary figure 5.

Uncropped Western blot used for the indicated figures

Supplementary table 1

NR	age	gender	Ig subtype	ISS stage (diagnosis)	DS stage (diagnosis)	time diagnosis > biopsy (months)	status at biopsy
1	79	m	IgAkappa	3	II A	0	untreated
2	54	m	IgA kappa	3	III A	12	relapsed/refractory
3	40	f	kappa	1	III A	47	relapsed/refractory
4	74	m	IgA lambda	2	I A	84	relapsed/refractory
5	71	f	IgG kappa	1	II A	31	relapsed/refractory
6	55	m	IgG kappa	3	III A	11	relapsed/refractory
7	80	f	IgG lambda	1	II A	31	relapsed/refractory
8	82	f	IgG lambda	3	III A	1	untreated
9	57	f	IgA kappa	2	III A	0	untreated
10	57	m	IgG kappa	1	II A	8	pretreated - 1st line
11	75	f	IgG lambda	n/a	II A	96	relapsed/refractory
12	53	f	lambda	3	III B	12	relapsed/refractory
13	60	m	IgA kappa	1	III A	25	pretreated - 1st line
14	55	f	IgA kappa	1	I A	50	relapsed/refractory

Abbreviations: alloPBSCT: allogeneic peripheral stem cell transplantation; **autoPBSCT:** autologous peripheral stem cell transplantation; **BB** [illegible] engager; **BEAM:** Carmustin/Etoposid/Cytarabin/Melphalan; **CarCyD:** Carfilzomib/Cyclophosphamid/Dexamethasone; **CE:** Cyclophosphamid [illegible] **Mel:** High dose Melphalan; **Ifo/MTX/VCR/Eto:** Ifosfamid/Metothrexat/Vincristin/Etoposid; **KRd:** Carfilzomib/Lenalidomide/Dexamethasone; **Le** [illegible] Lenalidomide/Doxorubicin/Dexamethasone; **Rd:** Lenalidomide/Dexamethasone; **RVCD:** Lenalidomid/ Bortezomib/Cyclophosphamid/Dexam [illegible] **VRD:** Bortezomib/Lenalidomide/Dexamethasone

Supplementary figure 1

a

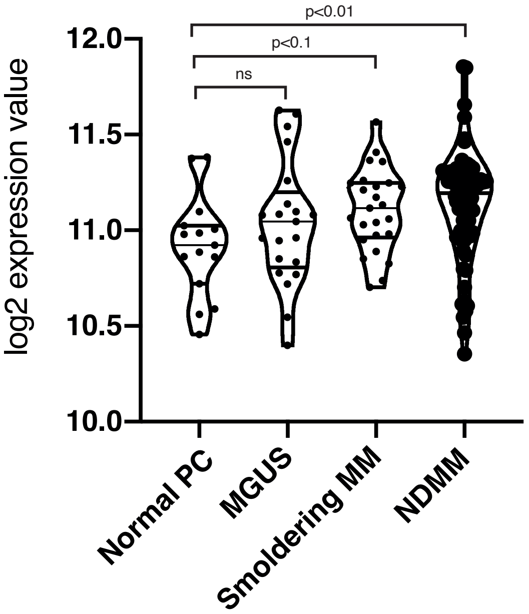

b

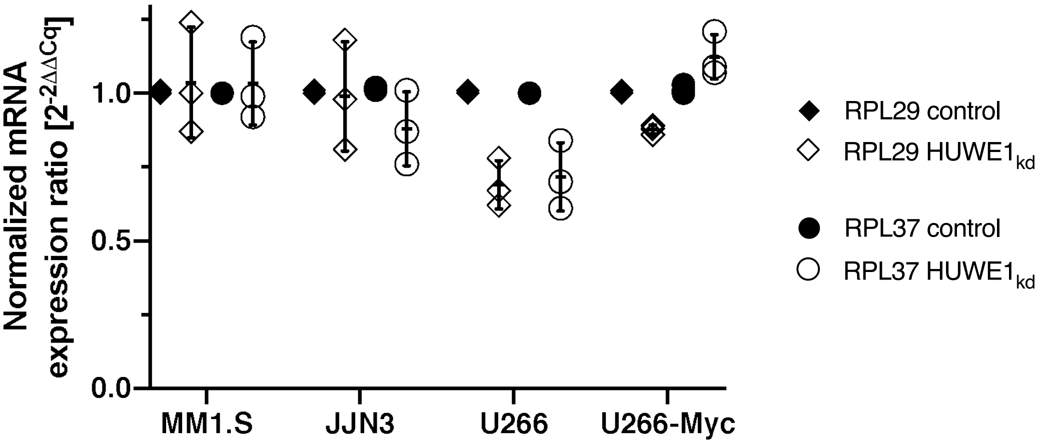

c

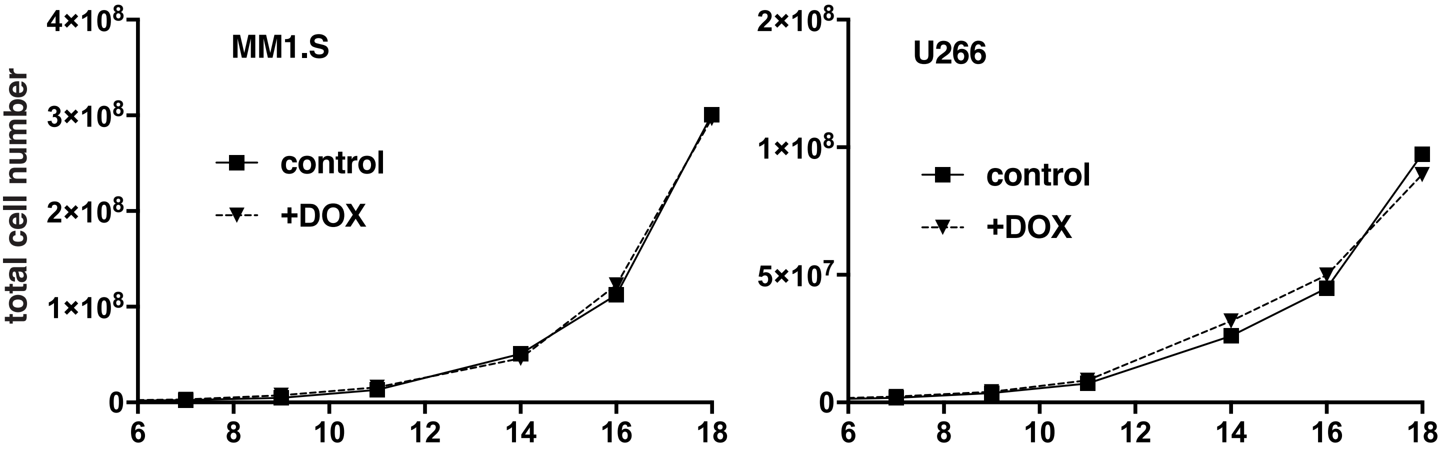

Supplementary figure 2

a

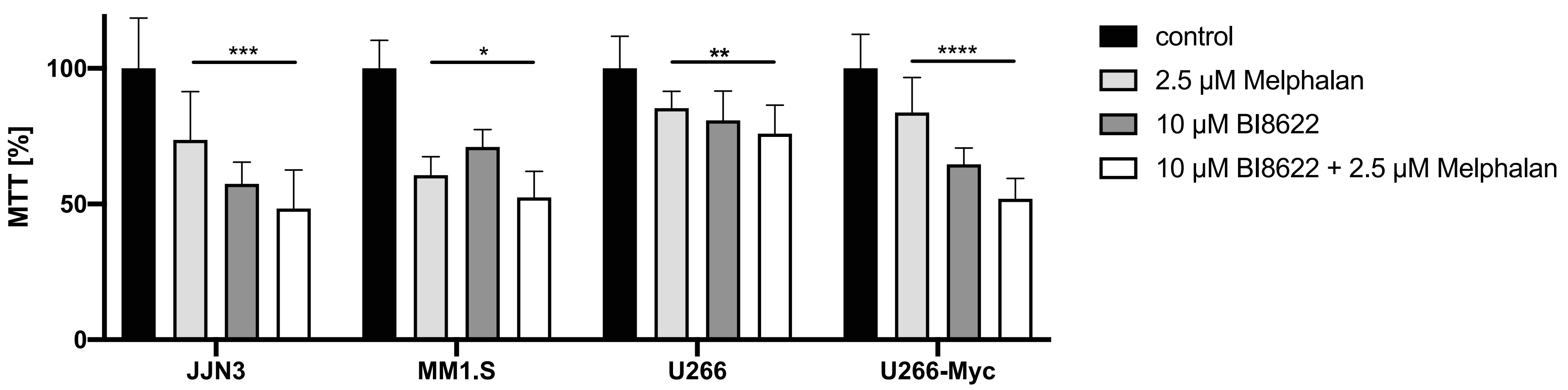

b

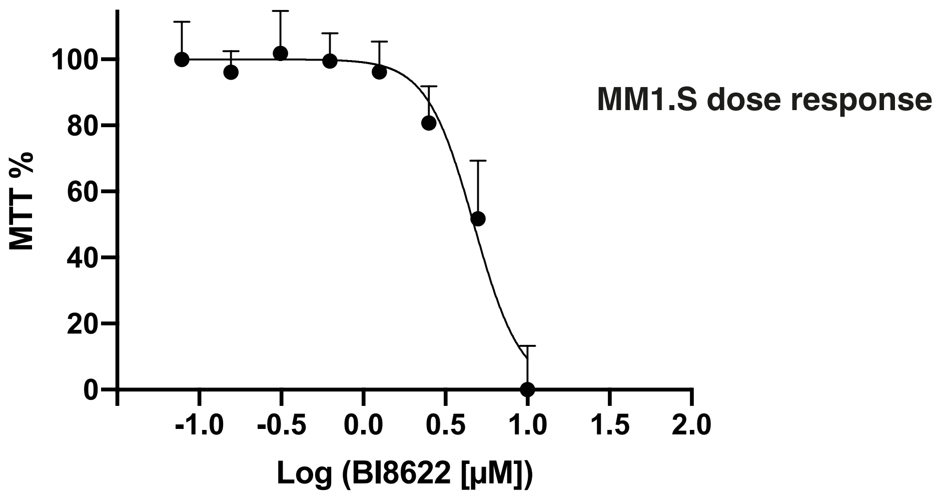

c

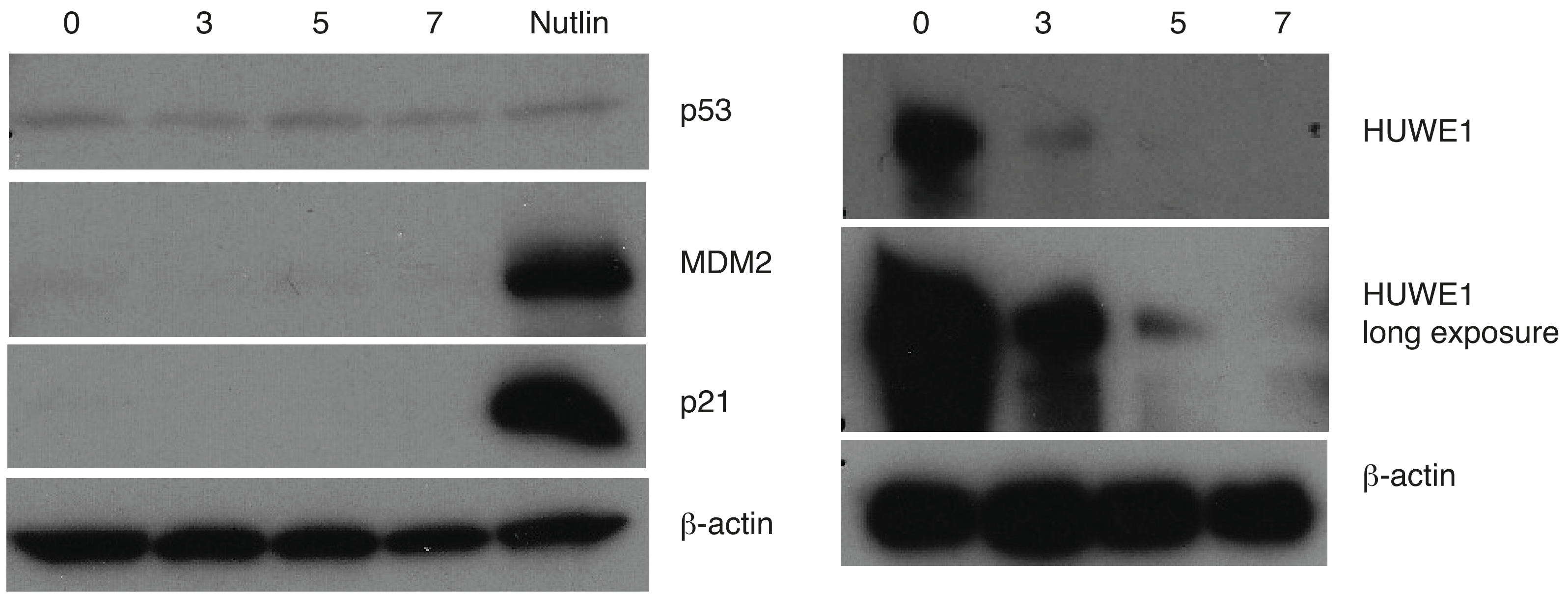

Supplementary figure 3

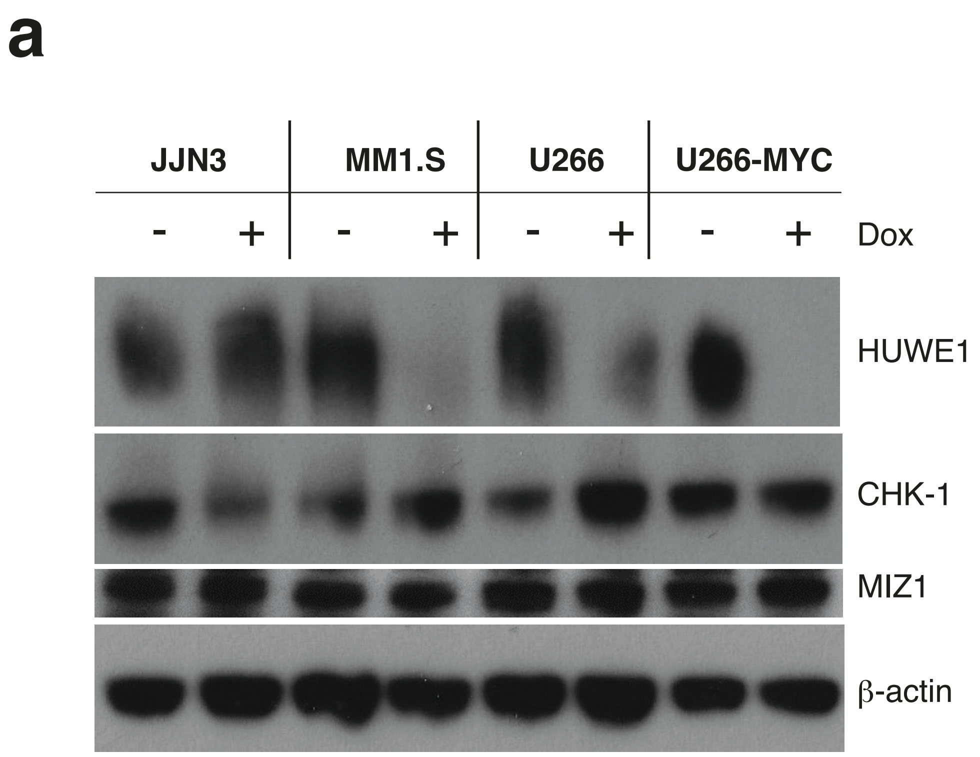

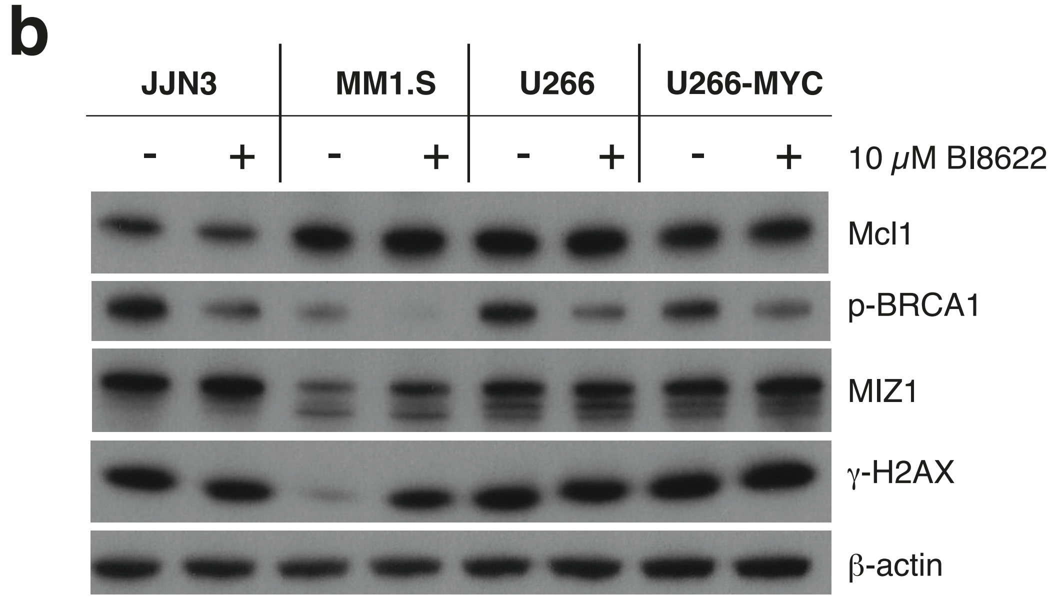

Supplementary figure 4

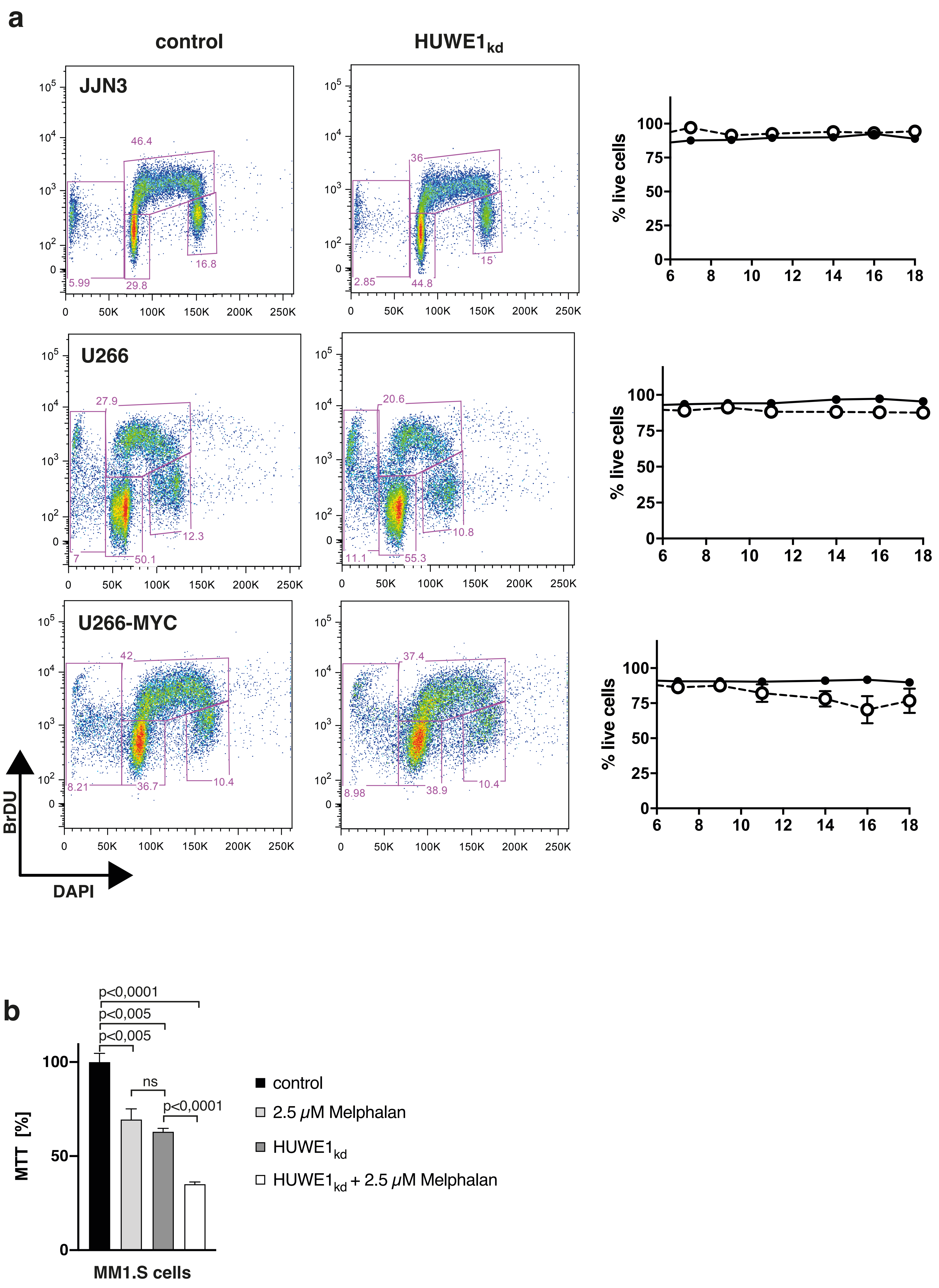

Supplementary figure 5

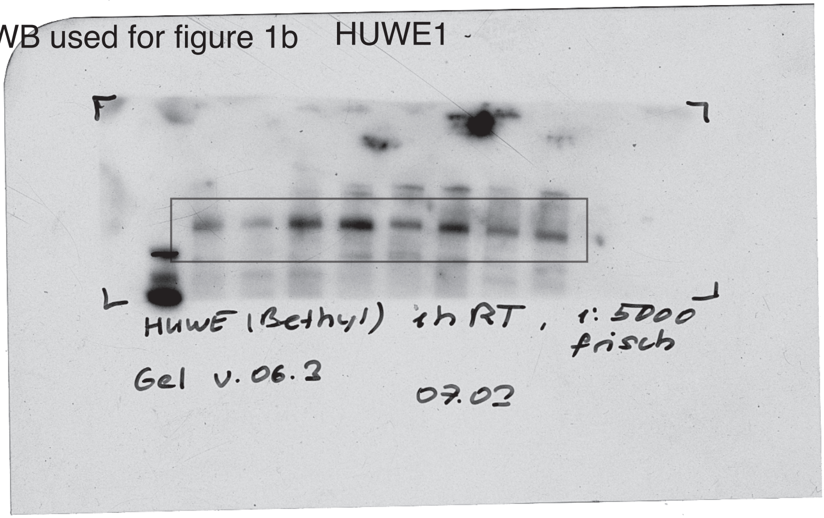

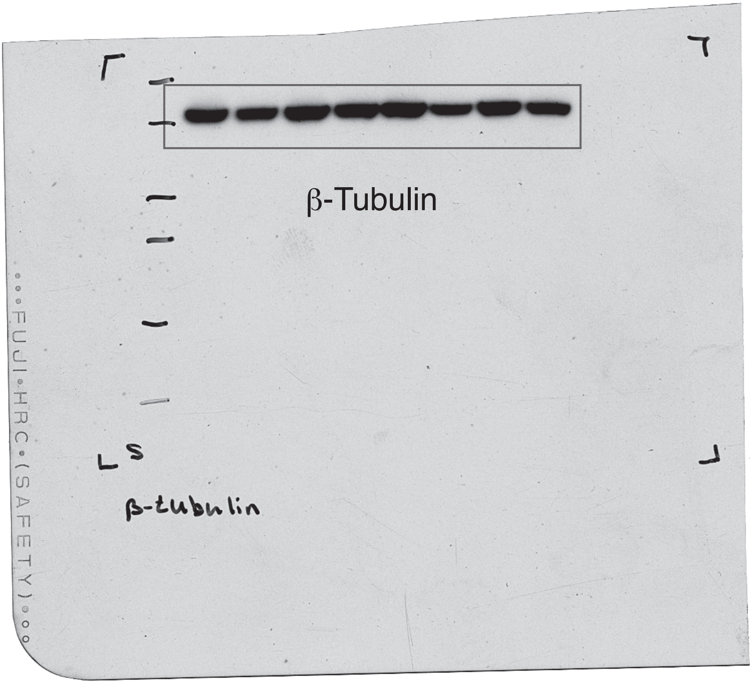

WB used for figure 2c

HUWE1

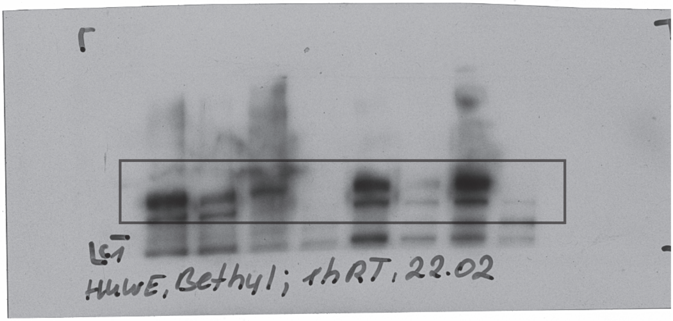

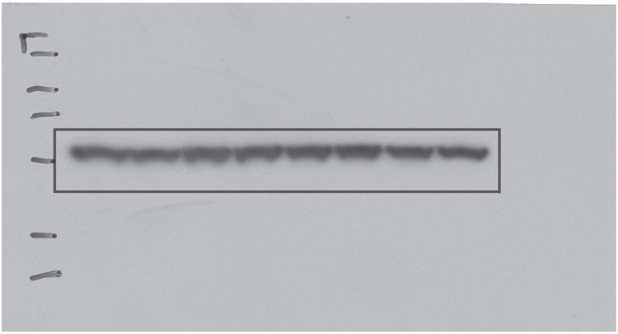

β-Tubulin

WB used for figure 4a

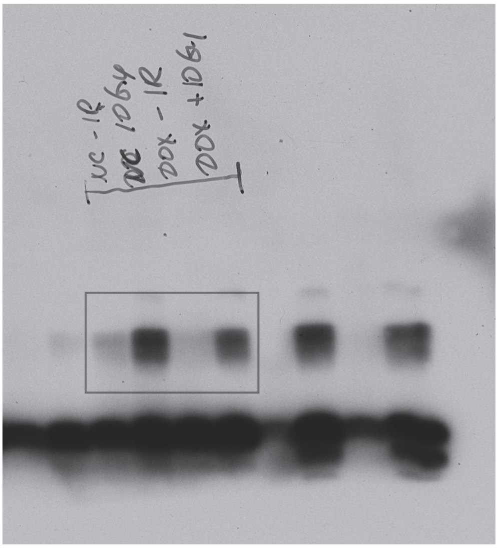

Ub-γH2AX

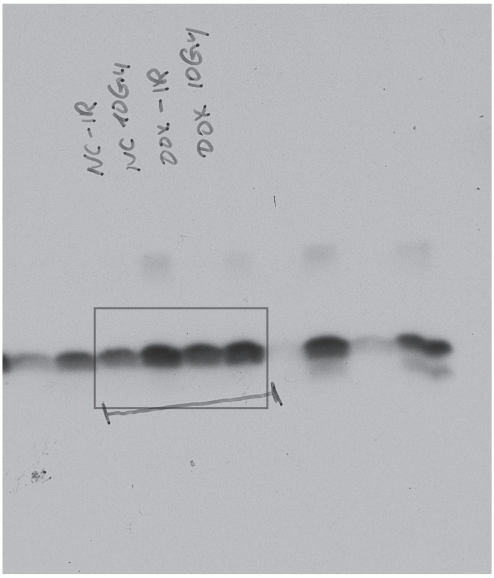

γH2AX

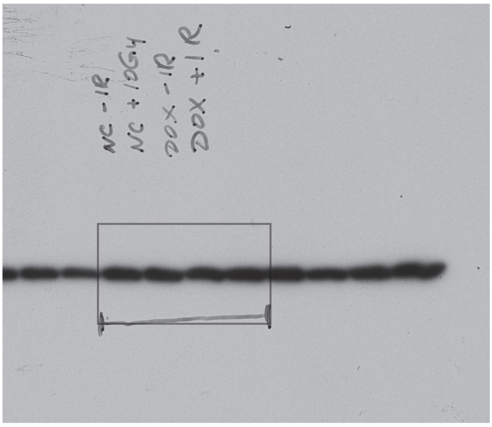

β–actin

WB used for figure 4d

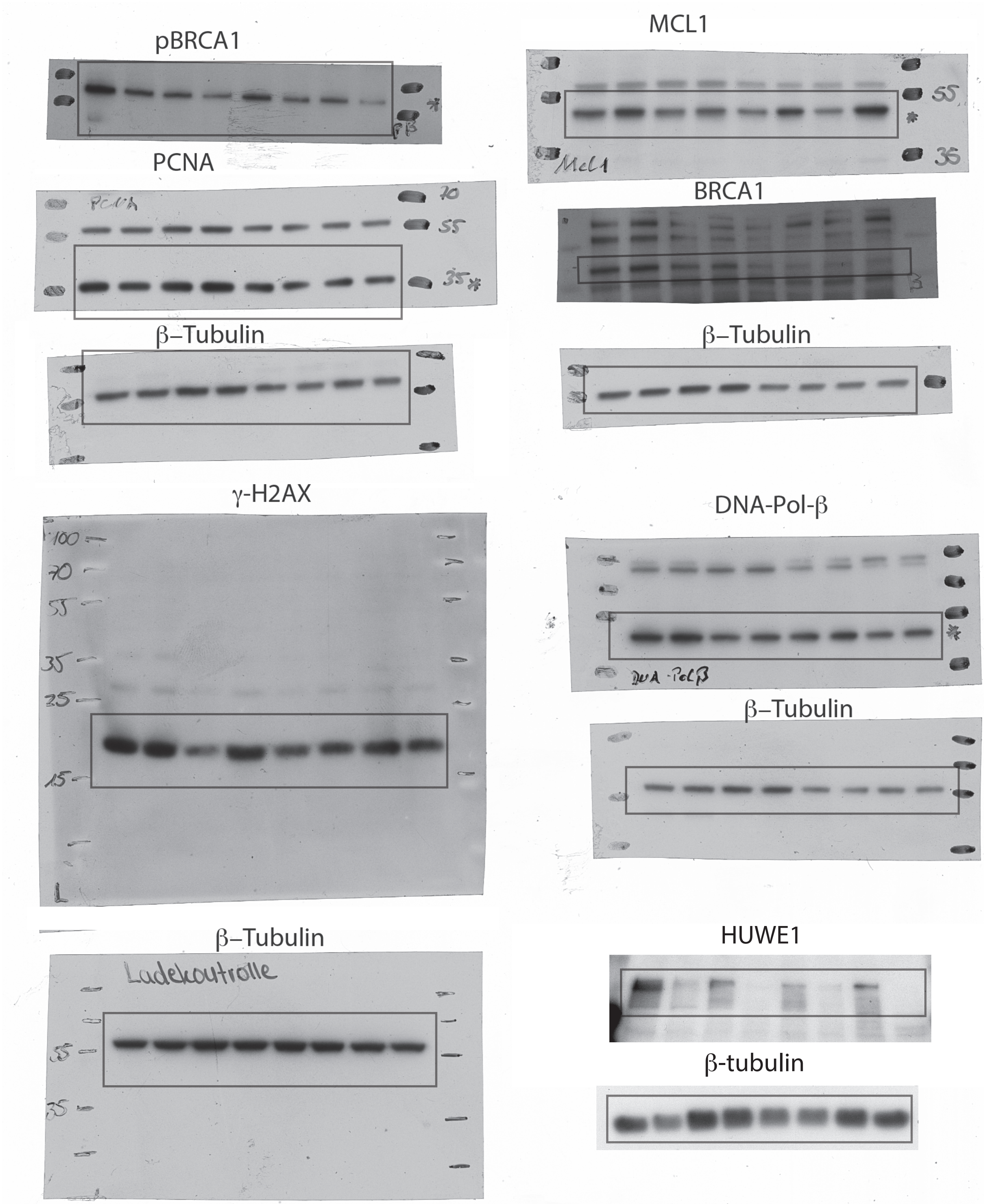

WB used for supplementary figure 2c

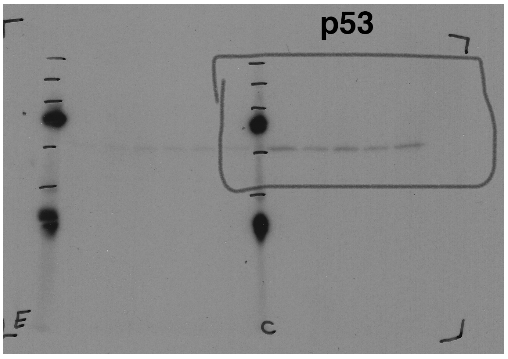

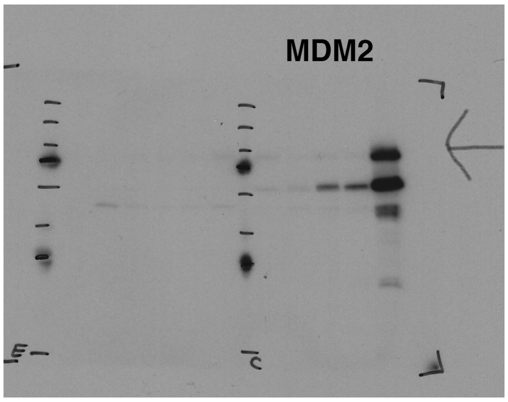

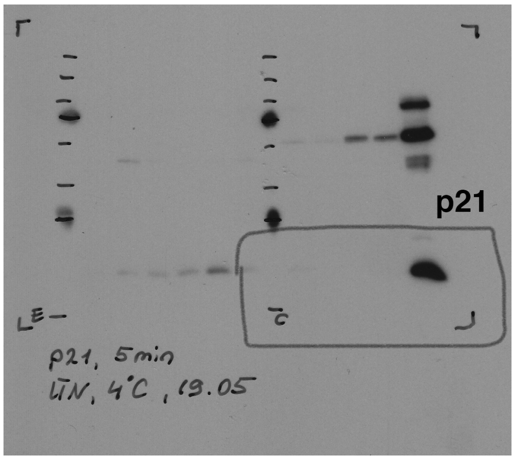

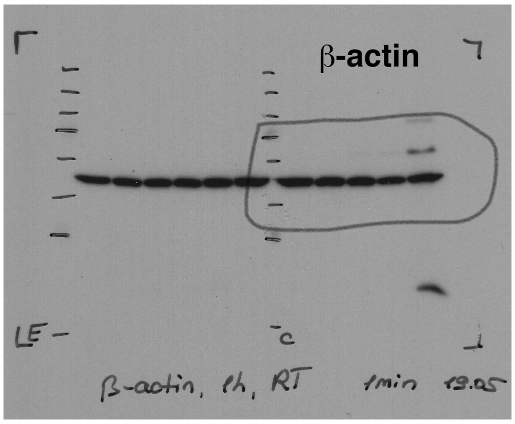

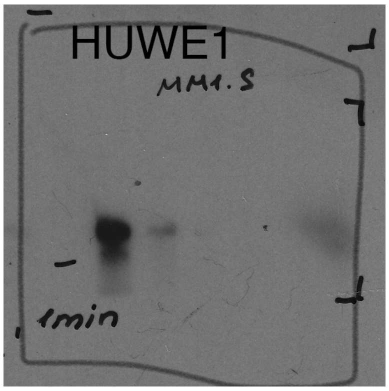

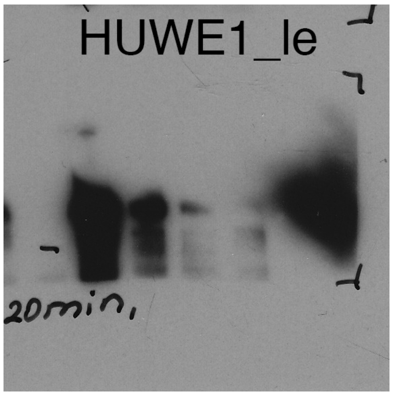

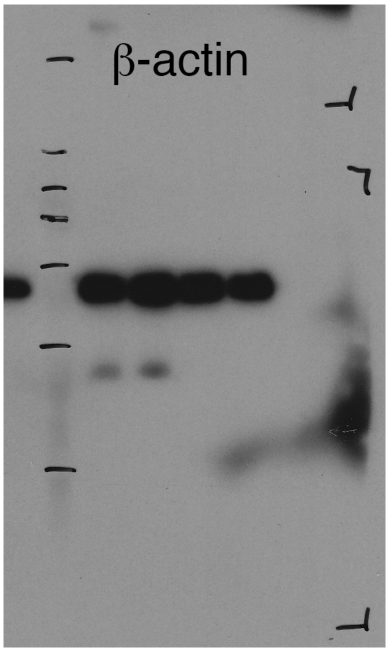

WB used for supplementary figure 3

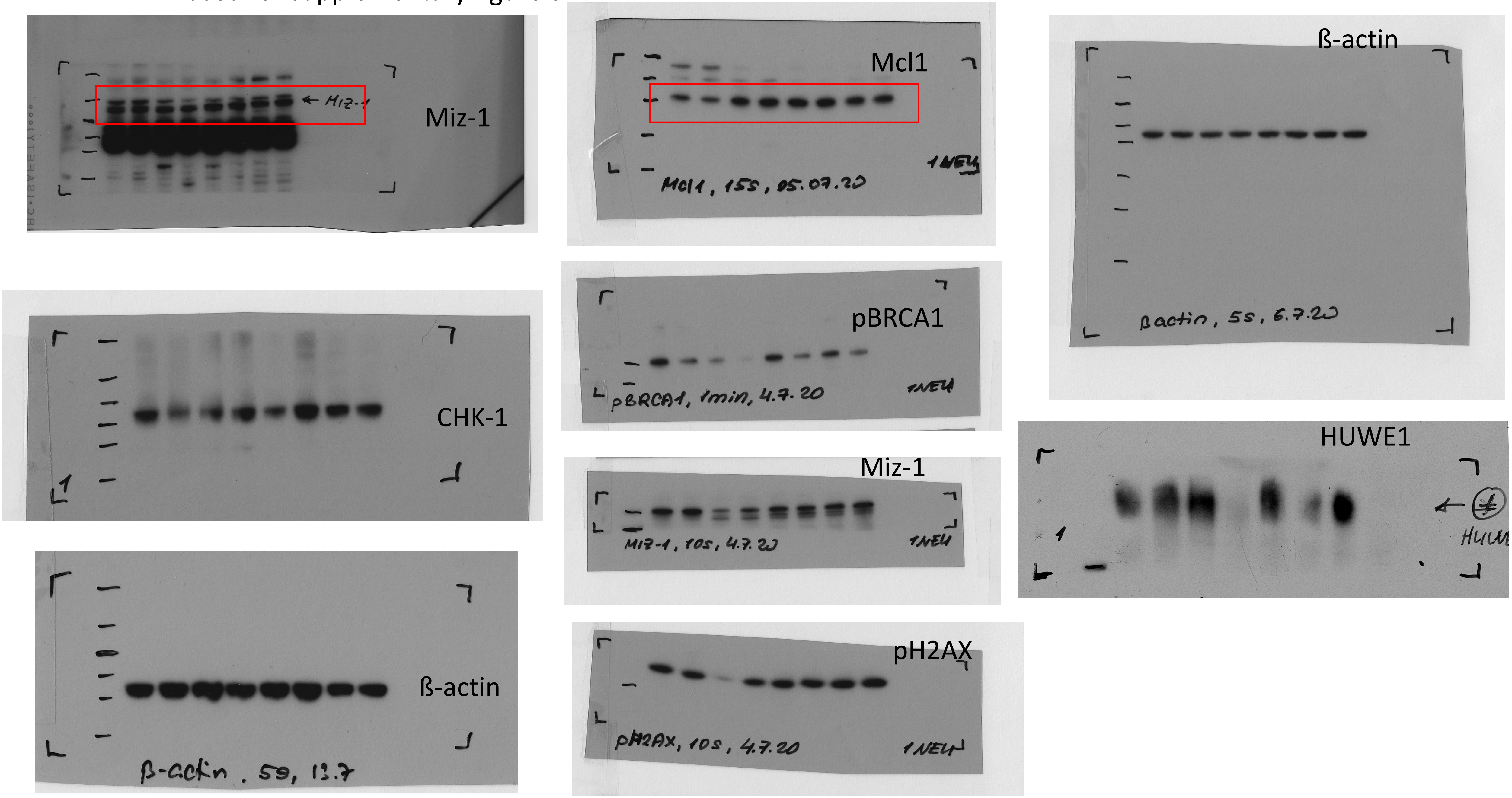

Supplement: Supplementary file 1 — Supplementary Information. [file 41598_2020_75499_MOESM1_ESM.pdf]
